# Supplementary material for: Clinical performance validation of the STANDARD G6PD test: A multi-country pooled analysis
Source: PLoS Negl Trop Dis. 2023 Oct 12;17(10):e0011652. doi: 10.1371/journal.pntd.0011652 (PMC10597494; doi:10.1371/journal.pntd.0011652)
Supplement: S1 Table — (DOCX) [file pntd.0011652.s001.docx]

**S1 Table. Summary of included studies.**

| **Country** | **Number of sites** | **Citation** | **Study type** | **Specimen type** | **Study population** | | **Description of venous specimen transport and storage conditions** | **Overseeing ethics committee(s) *Clinicaltrials.gov identifier, if applicable*** |
| --- | --- | --- | --- | --- | --- | --- | --- | --- |
|  |  |  |  |  | **Age** | **Description of sample** |  |  |
| Bangladesh | 1 | [21] | Prospective | Fresh venous K_2_EDTA | Not available | Individuals with known G6PD status from prior research studies | Specimens were collected in a K_2_EDTA vacutainer, stored at 4°C, and transported within 24-hours to the laboratory. Testing was conducted upon arrival. | Ethical review committee (ERC) and research review committee (RRC) of the icddr,b (PR-17043), the Australian Human Research Ethics Committee  (HREC) of the Northern Territory (HREC 17.2771) |
| Brazil | 2 | [20] | Prospective | Fresh capillary  Fresh venous K_2_EDTA | ≥2 years | Patients seeking care at clinics in Manaus and Porto Velho, and an enriched sample of participants with known G6PD status from prior research studies | Specimens were stored at 4-6°C immediately after collection and transferred to the laboratory. Reference testing was conducted within 72-hours of collection for spectrophotometry and 24-hours for hemoglobin. | PATH Research Ethics Committee (1204742), Ethics Committee Board of FMT/HVD (94833618.0.1001.0005), Brazil’s  National Research Ethics Commission (CONEP; 94833618.0.1001.0005), CEPEM ethics committee (94833618.0.2001.0011). NCT04033640 |
| Ethiopia | 2 | [23] | Prospective | Fresh capillary  Fresh venous K_2_EDTA | ≥2 years | Healthy participants | Specimens were stored at 4-6°C immediately after collection and transferred to the laboratory. Reference testing was conducted within 72-hours of collection for spectrophotometry and 24-hours for hemoglobin. | PATH Research Ethics Committee (1185779)  Ethiopian National Research Ethics Review Committee |
| India | 2 | [24] | Prospective | Fresh capillary  Fresh venous K_2_EDTA | ≥8 years | Febrile patients seeking care | Specimens were stored at 4-6°C after collection and transferred to the laboratory. Reference testing was conducted within 72-hours of collection for spectrophotometry and hemoglobin. | PATH Research Ethics Committee (1223628)  Medical College of Kolkata Institutional Ethics Committee,  Indian Council for Medical Research (ICMR)- National Institute of Cholera and Enteric Disease Institutional Ethics Committee |
| Thailand | 1 | [19] | Retrospective | Frozen venous K_2_EDTA | ≥18 years | Adults with known G6PD status | Specimens were stored at 4-6°C immediately after collection and transferred to the laboratory. Reference testing was conducted within 72 hours of collection for spectrophotometry and hemoglobin. | Mahidol University Faculty of Tropical Medicine (FTMEC MO/15/259) and the University of Oxford Tropical Research Ethics Committee (OXTREC 563-15) |
| United Kingdom | 1 | [22] | Retrospective | Fresh venous K_2_EDTA | ≥2 days | De-identified specimens from completed laboratory testing | Specimens were collected and stored at 4-6°C until reference testing, which was conducted within 7 days of collection | Non-research determination from the PATH Research Determination Committee |
| United States | 3 | [22] | Prospective | Fresh capillary  Fresh venous K_2_EDTA | ≥18 years | Healthy adults | Specimens were stored at 4-6°C immediately after collection and shipped over night on ice pack at PATH and UW laboratories. Reference testing was conducted within 48-hours of collection for spectrophotometry and hemoglobin. | Florida: Western Institutional Review Board (20161665)  Pennsylvania: PATH Research Ethics Committee (1416844)  Washington: Fred Hutchinson Cancer Research Center institutional review board [IRB] (10091)  Pennsylvania: NCT04054661  Washington: NCT04010695 |
| United States | 1 | [19] | Prospective | Fresh venous K_2_EDTA | ≥18 years | African American blood donors from sites in New York and Miami | Specimens were collected, shipped and stored at 4-6°C until reference testing. All testing was completed within 4 days of collection. | Western Institutional Review Board (20161665) |
| Contrived specimen panel | 1 | [22] | Contrived | Fresh venous K_2_EDTA | N/A | | Specimens were collected, shipped and stored at 4-6°C until testing. Specimens were split into two aliquots: one aliquot was refrigerated, and the other aliquot was incubated in a temperature-controlled water bath. Both aliquots were used appropriately to create the contrived panel. All testing was completed within 24-hours of sample panel generation. | N/A |
