# Supplementary material for: Clinical performance validation of the STANDARD G6PD test: A multi-country pooled analysis
Source: PLoS Negl Trop Dis. 2023 Oct 12;17(10):e0011652. doi: 10.1371/journal.pntd.0011652 (PMC10597494; doi:10.1371/journal.pntd.0011652)
Supplement: S2 Table — (DOCX) [file pntd.0011652.s002.docx]

**S2 Table. Hemoglobin ranges for anemia (g/dL)**

| **Population** | **Non-anemia** | **Anemia** | | |
| --- | --- | --- | --- | --- |
|  |  | **Mild** | **Moderate** | **Severe** |
| Children 6-59 months of age | ≥ 11 | 10.0–10.9 | 7.0–9.9 | < 7.0 |
| Children 5-11 years of age | ≥ 11.5 | 11.0–11.4 | 8.0–10.9 | < 8.0 |
| Children 12-14 years of age | ≥ 12 | 11.0–11.9 | 8.0–10.9 | < 8.0 |
| Non-pregnant women (15 years of age and above) | ≥ 12 | 11.0–11.9 | 8.0–10.9 | < 8.0 |
| Pregnant women | ≥ 11 | 10.0–10.9 | 7.0–9.9 | < 7.0 |
| Men (15 years of age and above) | ≥ 13 | 11.0–12.9 | 8.0–10.9 | < 8.0 |

Source: World Health Organization. *Haemoglobin Concentrations for the Diagnosis of Anaemia and Assessment of Severity*. World Health Organization; 2011. Accessed March 15, 2021. <https://www.who.int/vmnis/indicators/haemoglobin.pdf>
