# Supplementary material for: Clinical performance validation of the STANDARD G6PD test: A multi-country pooled analysis
Source: PLoS Negl Trop Dis. 2023 Oct 12;17(10):e0011652. doi: 10.1371/journal.pntd.0011652 (PMC10597494; doi:10.1371/journal.pntd.0011652)
Supplement: S3 Table — (DOCX) [file pntd.0011652.s003.docx]

**S3 Table. STANDARD G6PD Test result classifications and WHO recommendations for treatment eligibility with Primaquine and the label indications for Tafenoquine.**

|  | **STANDARD G6PD Test Result** | **G6PD classification or percent activity** | **Recommended or eligible for treatment with primaquine or tafenoquine** |
| --- | --- | --- | --- |
| Primaquine eligibility | Males and females ≤ 4.0 U/g Hb | G6PD deficient | Consider preventing relapse by giving PQ once a week with medical supervision* |
|  | Females 4.1 – 6.0 U/g Hb | G6PD intermediate | Eligible for the 14-day regimen of primaquine, with counselling on how to recognize symptoms and signs of hemolytic anemia |
|  | Males > 4.0 U/g Hb  Females > 6.0 U/g Hb | G6PD normal | Treat in children and adults with a 14-day course in all transmission settings ** |
|  | N/A | G6PD status unknown or testing unavailable | Decision to prescribe PQ based on an assessment of risks and benefits*** |
| Tafenoquine eligibility | Males and females ≤ 6.0 U/g Hb | ≤ 70% | Ineligible for tafenoquine |
|  | Males and females > 6.0 U/g Hb | > 70% | Eligible for tafenoquine **** |
|  | N/A | G6PD status unknown or testing unavailable | Ineligible for tafenoquine |

**Note, this regimen is not commonly prescribed in practice and for the purposes of this analysis, those testing as G6PD deficient are considered to not have access to PQ or TQ*

***Except pregnant women, infants aged < 6 months, women breastfeeding infants aged < 6 months, women breastfeeding older infants unless they are known not to be G6PD deficient and people with G6PD deficiency.*

**** Risks include low relapse rates, low P. vivax incidence rates, high G6PD deficiency prevalence, patient unable to detect signs and symptoms of hemolysis, and patient has poor access to health care system*

***** Eligibility is also currently restricted to those older than 16 years of age, though pediatric studies are ongoing*

Source: World Health Organization (WHO). Guide to G6PD deficiency rapid diagnostic testing to support P. vivax radical cure. Published online 2018. Licence: CC BY-NC-SA 3.0 IGO.
