# Supplementary material for: Clinical performance validation of the STANDARD G6PD test: A multi-country pooled analysis
Source: PLoS Negl Trop Dis. 2023 Oct 12;17(10):e0011652. doi: 10.1371/journal.pntd.0011652 (PMC10597494; doi:10.1371/journal.pntd.0011652)
Supplement: S4 Table — (DOCX) [file pntd.0011652.s004.docx]

**S4 Table . Site-specific adjusted male medians (AMMs) used for reference-assay threshold determination.**

|  | **Bangladesh^a^** | **Brazil^b^** | | **Ethiopia^c^** | | **India^d^** | **UK^e^** | **US (2021)^e^** | | **US (2019)^f^** | **Thailand^f^** |
| --- | --- | --- | --- | --- | --- | --- | --- | --- | --- | --- | --- |
|  |  | **Manaus** | **Porto Velho** | **Jimma** | **Gambella** |  |  | **Pennsylvania, Washington, and Contrived specimens** | **Florida** |  |  |
| Median 100% | 9.9 | 8.63 | 8.93 | 8.11 | 7.89 | 8.56 | 8.7 | 12.85 | 7.49 | 9.03 | 6.84 |
| 30% | 2.97 | 2.58 | 2.67 | 2.43 | 2.37 | 2.57 | 2.61 | 3.87 | 2.25 | 2.71 | 2.05 |
| 70% | 6.93 | 6.02 | 6.23 | 5.67 | 5.52 | 5.99 | 6.09 | 9.03 | 5.24 | 6.32 | 4.79 |

a. Data published within [21].

b .Data published within [20].

c. Data published at [23].

d. Data published at [24].

e. Data published within [22].

f. Data published within [19].
