# Supplementary material for: Clinical performance validation of the STANDARD G6PD test: A multi-country pooled analysis
Source: PLoS Negl Trop Dis. 2023 Oct 12;17(10):e0011652. doi: 10.1371/journal.pntd.0011652 (PMC10597494; doi:10.1371/journal.pntd.0011652)
Supplement: S5 Table — (DOCX) [file pntd.0011652.s005.docx]

**S5 Table. Median G6PD values and interquartile ranges from all normal males in the analytical populations*, by site***

|  | **Bangladesh^a^** | **Brazil^b^** | **Ethiopia^c^** | **India^d^** | **UK^e^** | **US (2021)^e^** | **US (2019)^f^** | **Thailand^f^** |
| --- | --- | --- | --- | --- | --- | --- | --- | --- |
| G6PD median (IQR) Pointe Scientific, venous specimens  (U/g Hb) | **8.3**  (8.1– 9.1) | **9.0**  (8.3 – 9.7) | **8.1**  (7.2 – 9.0) | **8.7**  (7.7 -10.0) | **11.5**  (10.2 -14.2) | **11.9**  (10.4 – 13.5) | **9.7**  (8.1 – 10.9) | **6.8**  (6.6 – 7.8) |
| G6PD median (IQR) STANDARD G6PD Test, capillary specimens (U/g Hb) | N/A | **7.5**  (6.6 – 8.4) | **7.6**  (6.8– 8.3) | **8.5**  (7.6 – 9.9) | N/A | **7.6**  (6.7 – 8.8) | N/A | N/A |
| G6PD median (IQR) STANDARD G6PD Test, venous specimens (U/g Hb) | **7.9**  (6.9 – 8.8) | **7.9**  (7.1 – 8.8) | **7.9**  (7.1 – 8.8) | **8.1**  (7.1 – 9.5) | **11.3**  (9.4 – 13.9) | **7.3**  (6.5 – 8.4) | **10.1**  (8.5 – 11.6) | **6.7**  (5.7 – 7.7) |

Abbreviation: IQR, interquartile range.

* Where applicable, excluding data from normal males that were used to calculate the adjusted male medians (AMM).

a. Data published within [21].

b .Data published within [20].

c. Data published at Domingo [23].

d. Data published at Domingo [24].

e. Data published within [22].

f. Data published within [19].
