# Supplementary material for: Clinical performance validation of the STANDARD G6PD test: A multi-country pooled analysis
Source: PLoS Negl Trop Dis. 2023 Oct 12;17(10):e0011652. doi: 10.1371/journal.pntd.0011652 (PMC10597494; doi:10.1371/journal.pntd.0011652)
Supplement: S6 Table — (DOCX) [file pntd.0011652.s006.docx]

**S6 Table. Contingency tables showing agreement in case counts across the STANDARD G6PD Test and the reference assay using the manufacturer’s threshold values at 30% and 70% G6PD activity thresholds, on A) capillary specimens, B) venous specimens (excluding contrived), and C) Venous specimens (including contrived).**

1. Capillary

|  | | **G6PD cases defined by the reference assay** | | | **Total** |
| --- | --- | --- | --- | --- | --- |
|  |  | **Deficient** | **Intermediate** | **Normal** |  |
| **STANDARD G6PD Test** | **Deficient** | 143 | 30 | 49 | 222 |
|  | **Intermediate** | 0 | 37 | 120 | 157 |
|  | **Normal** | 0 | 20 | 3,813 | 3,833 |
|  | **Total** | 143 | 87 | 3,982 | 4,212 |

Percent agreement between hemoglobin-normalized G6PD activity categorized results and the STANDARD G6PD Test was 94.8% [95% CI: 94.1–95.5].

Kappa: 0.69

1. Venous (excluding contrived)

|  | | **G6PD cases defined by the reference assay** | | | **Total** |
| --- | --- | --- | --- | --- | --- |
|  |  | **Deficient** | **Intermediate** | **Normal** |  |
| **STANDARD G6PD Test** | **Deficient** | 259 | 69 | 41 | 369 |
|  | **Intermediate** | 0 | 80 | 113 | 193 |
|  | **Normal** | 0 | 23 | 4,164 | 4,187 |
|  | **Total** | 259 | 172 | 4,318 | 4,749 |

Percent agreement between hemoglobin-normalized G6PD activity categorized results and the STANDARD G6PD Test was 94.8% [95% CI: 94.2–95.4].

Kappa: 0.69

1. Venous (including contrived)

|  | | **G6PD cases defined by the reference assay** | | | **Total** |
| --- | --- | --- | --- | --- | --- |
|  |  | **Deficient** | **Intermediate** | **Normal** |  |
| **STANDARD G6PD Test** | **Deficient** | 262 | 111 | 41 | 414 |
|  | **Intermediate** | 0 | 96 | 125 | 221 |
|  | **Normal** | 0 | 23 | 4,186 | 4,209 |
|  | **Total** | 262 | 230 | 4,352 | 4,844 |

Percent agreement between hemoglobin-normalized G6PD activity categorized results and the STANDARD G6PD Test was 93.8% [95% CI: 93.1–94.5].

Kappa: 0.69
