# Supplementary material for: Clinical performance validation of the STANDARD G6PD test: A multi-country pooled analysis
Source: PLoS Negl Trop Dis. 2023 Oct 12;17(10):e0011652. doi: 10.1371/journal.pntd.0011652 (PMC10597494; doi:10.1371/journal.pntd.0011652)
Supplement: S7 Table — (DOCX) [file pntd.0011652.s007.docx]

**S7 Table. Descriptive statistics of the operating temperatures (degrees Celsius) and humidities (%) under which the STANDARD G6PD Test was run, by specimen type.**

|  | Capillary | Venous |
| --- | --- | --- |
| Temperature |  |  |
| Mean temperature (SD) | 24.9 (3.4) | 24.8 (2.8) |
| Median temperature | 24.2 | 24.1 |
| Range | 17.1 – 43.7 | 18.5 – 33.3 |
| Humidity |  |  |
| Mean humidity (SD) | 59.5 (11.0) | 53.2 (11.9) |
| Median humidity | 59.0 | 52.2 |
| Range | 10.0 – 93.7 | 7.1 – 92.0 |
