# Supplementary material for: Clinical performance validation of the STANDARD G6PD test: A multi-country pooled analysis
Source: PLoS Negl Trop Dis. 2023 Oct 12;17(10):e0011652. doi: 10.1371/journal.pntd.0011652 (PMC10597494; doi:10.1371/journal.pntd.0011652)
Supplement: S8 Table — (DOCX) [file pntd.0011652.s008.docx]

**S8 Table. Areas under the curve (AUC) for receiver operating characteristics (ROC) analysis of the performance of the STANDARD G6PD Test for G6PD activity against the reference test for G6PD-deficient males and females as well as intermediate females, by specimen type.**

| **STANDARD G6PD activity** | **AUC** |
| --- | --- |
| **Capillary specimens** | |
| 30% activity males and females | 0.998 |
| 70% activity females only | 0.909 |
| **Venous specimens** | |
| 30% activity males and females | 0.997 |
| 70% activity females only | 0.954 |
