# Supplementary material for: Clinical performance validation of the STANDARD G6PD test: A multi-country pooled analysis
Source: PLoS Negl Trop Dis. 2023 Oct 12;17(10):e0011652. doi: 10.1371/journal.pntd.0011652 (PMC10597494; doi:10.1371/journal.pntd.0011652)
Supplement: S9 Table — (DOCX) [file pntd.0011652.s009.docx]

**S9 Table. Sensitivity analysis for diagnostic performance on a) capillary, and b) venous specimens.**

**A. Capillary**

|  | **Number of cases** | | | **Performance for G6PD deficient males and females** | | | | **Performance for G6PD intermediate females (> 30, ≤ 70%)** | | | |
| --- | --- | --- | --- | --- | --- | --- | --- | --- | --- | --- | --- |
|  | **Deficient** | **Intermediate** | **Normal** | **Sensitivity**  **(95% CI)** | **Specificity**  **(95% CI)** | **PPV**  **(95% CI)** | **NPV**  **(95%CI)** | **Sensitivity**  **(95% CI)** | **Specificity**  **(95% CI)** | **PPV**  **(95% CI)** | **NPV**  **(95%CI)** |
| Pooled, all studies | 143 | 79 | 3990 | 100.0  (97.5–100.0) | 98.1  (97.6–98.5) | 64.4  (57.7–70.7) | 100.0  (99.9–100.0) | 77.0  (66.8–85.4) | 92.8  (91.6–93.9) | 32.5  (26.2–39.4) | 98.9  (98.3–99.3) |
| Pooled, without Brazil^b^ | 85 | 43 | 2391 | 100.0  (95.8–100.0) | 98.2  (97.6–98.7) | 66.4  (57.5–74.5) | 100.0  (99.8–100.0) | 65.4  (50.9–78.0) | 93.2  (91.6–94.7) | 32.4  (23.6–42.2) | 98.2  (97.2–98.9) |
| Pooled, without Ethiopia^c^ | 131 | 72 | 3000 | 100.0  (97.2–100.0) | 97.7  (97.1–98.2) | 64.5  (57.5–71.1) | 100.0  (99.9–100.0) | 83.8  (72.9–91.6) | 92.2  (90.7–93.5) | 32.9  (26.0–40.5) | 99.2  (98.6–99.6) |
| Pooled, without India^d^ | 116 | 58 | 3149 | 100.0  (96.9–100.0) | 98.2  (97.7–98.6) | 66.7  (59.1–73.6) | 100.0  (99.9–100.0) | 80.3  (69.1–88.8) | 92.2  (90.8–93.4) | 31.0  (24.4–38.2) | 99.1  (98.5–99.5) |
| Pooled, without US (2021)^e^ | 97 | 64 | 3430 | 100.0  (96.3–100.0) | 98.2  (97.7–98.6) | 60.2  (52.2–67.9) | 100.0  (99.9–100.0) | 75.7  (64.0–85.2) | 93.7  (92.4–94.9) | 34.0  (26.6–42.0) | 98.9  (98.3–99.4) |

**B. Venous**

|  | **Number of cases** | | | **Performance for G6PD deficient males and females** | | | | **Performance for G6PD intermediate females (> 30, ≤ 70%)** | | | |
| --- | --- | --- | --- | --- | --- | --- | --- | --- | --- | --- | --- |
|  | **Deficient** | **Intermediate** | **Normal** | **Sensitivity**  **(95% CI)** | **Specificity**  **(95% CI)** | **PPV**  **(95% CI)** | **NPV**  **(95%CI)** | **Sensitivity**  **(95% CI)** | **Specificity**  **(95% CI)** | **PPV**  **(95% CI)** | **NPV**  **(95%CI)** |
| Pooled, all studies | 262 | 152 | 4430 | 100.0  (98.6–100.0) | 96.7  (96.1–97.2) | 63.3  (58.4–67.9) | 100.0  (99.9–100.0) | 89.0  (85.4–93.6) | 93.9  (92.8–94.9) | 61.1  (55.6–66.3) | 98.9  (98.3–99.3) |
| Pooled, without Bangladesh^a^ | 232 | 142 | 4362 | 100.0  (98.4–100.0) | 96.8  (96.3–97.3) | 62.0  (56.9–67.0) | 100.0  (99.9–100.0) | 90.0  (85.1–93.7) | 94.2  (93.1–95.1) | 60.3  (54.6–65.7) | 99.0  (98.4–99.4) |
| Pooled, without Brazil^b^ | 206 | 129 | 2847 | 100.0  (98.2–100.0) | 95.7  (94.9–96.4) | 61.5  (56.0–66.7) | 100.0  (99.9–100.0) | 88.9  (83.7–92.9) | 92.2  (90.6–93.6) | 63.5  (57.6–69.2) | 98.2  (97.3–98.9) |
| Pooled, without Ethiopia^c^ | 250 | 146 | 3433 | 100.0  (98.5–100.0) | 95.9  (95.2–96.5) | 63.1  (58.2–67.9) | 100.0  (99.9–100.0) | 93.4  (89.1–96.3) | 93.3  (92.0–94.4) | 63.1  (57.5–68.5) | 99.1  (98.6–99.5) |
| Pooled, without India^d^ | 236 | 132 | 3616 | 100.0  (98.4–100.0) | 96.5  (95.8–97.0) | 64.1  (59.0–69.0) | 100.0  (99.9–100.0) | 92.0  (87.5–95.3) | 93.9  (92.7–94.9) | 63.2  (57.6–68.6) | 99.0  (98.5–99.4) |
| Pooled, without UK^e^ | 252 | 143 | 4282 | 100.0  (98.5–100.0) | 96.8  (96.2–97.3) | 63.8  (58.8–68.5) | 100.0  (99.9–100.0) | 89.5  (84.7–93.3) | 93.8  (92.6–94.8) | 60.1  (54.5–65.4) | 98.8  (98.3–99.3) |
| Pooled, without contrived^e^ | 259 | 110 | 4380 | 100.0  (98.6–100.0) | 97.6  (97.1–98.0) | 70.2  (65.2–74.8) | 100.0  (99.9–100.0) | 86.6  (80.6–91.3) | 94.4  (93.3–95.3) | 55.4  (49.2–61.4) | 98.9  (98.3–99.3) |
| Pooled, without US (2021)^e**^ | 216 | 135 | 3880 | 100.0  (98.3–100.0) | 96.6  (96.0–97.2) | 61.5  (56.2–66.7) | 100.0  (99.9–100.0) | 89.7  (84.8–93.4) | 94.8  (93.7–95.8) | 66.1  (60.3–71.5) | 98.8  (98.2–99.2) |
| Pooled, without US (2019)^f**^ | 237 | 150 | 4283 | 100.0  (98.5–100.0) | 96.6  (96.0–97.1) | 61.2  (56.2–66.1) | 100.0  (99.9–100.0) | 90.5  (85.8–94.0) | 93.8  (92.6–94.7) | 60.1  (54.6–65.4) | 99.0  (98.4–99.3) |
| Pooled, without Thailand^f^ | 208 | 129 | 4357 | 100.0  (98.2–100.0) | 97.1  (96.6–97.6) | 61.7  (56.3–66.9) | 100.0  (99.9–100.0) | 88.6  (83.1–92.8) | 94.4  (93.3–95.3) | 57.4  (51.4–63.2) | 99.0  (98.4–99.4) |

a. Data published within [21].

b .Data published within [20].

c. Data published at [23].

d. Data published at [24].

e. Data published within [22].

f. Data published within [19].

** Florida, Pennsylvania, and Washington sites.
