# Supplementary material for: Clinical performance validation of the STANDARD G6PD test: A multi-country pooled analysis
Source: PLoS Negl Trop Dis. 2023 Oct 12;17(10):e0011652. doi: 10.1371/journal.pntd.0011652 (PMC10597494; doi:10.1371/journal.pntd.0011652)
Supplement: S10 Table — (DOCX) [file pntd.0011652.s010.docx]

**S10 Table. Summary of discordant false negative results on A) Capillary specimens and B) Venous specimens*.**

1. Capillary

| **Participant identifier** | **Site** | **Reference % G6PD activity** | **Capillary STANDARD G6PD Test G6PD Result (U/g Hb)** |
| --- | --- | --- | --- |
| ETGA-1037 | Ethiopia^c^ | 42 % | 8.5 |
| INCC-0256 | India^d^ | 49 % | 7.0 |
| INMC-0504 | India^d^ | 50 % | 8.1 |
| INMC-0505 | India^d^ | 56 % | 11.1 |
| ETGA-1220 | Ethiopia^c^ | 57 % | 9.2 |
| ETGA-1034 | Ethiopia^c^ | 58 % | 7.5 |
| ETGA-1285 | Ethiopia^c^ | 60 % | 6.4 |
| ETGA-0981 | Ethiopia^c^ | 60 % | 6.2 |
| INMC-0490 | India^d^ | 61 % | 6.2 |
| INMC-0509 | India^d^ | 63 % | 9.8 |
| INCC-0409 | India^d^ | 63 % | 8.8 |
| USPA159 | US^e^ | 64 % | 6.2 |
| ETGA-1331 | Ethiopia^c^ | 66 % | 6.9 |
| G243 | US^e^ | 67 % | 7.1 |
| BRMA-0667 | Brazil^b^ | 67 % | 6.7 |
| USPA206 | US^e^ | 67 % | 6.5 |
| ETGA-1221 | Ethiopia^c^ | 69 % | 8.0 |
| BRMA-0015 | Brazil^b^ | 69 % | 7.4 |
| ETGA-0985 | Ethiopia^c^ | 69 % | 6.8 |
| ETGA-1289 | Ethiopia^c^ | 69 % | 7.3 |

1. Venous*

| **Participant identifier** | **Site** | **Reference % G6PD activity** | **Venous STANDARD G6PD Test G6PD Result (U/g Hb)** |
| --- | --- | --- | --- |
| ETGA-1037 | Ethiopia^c^ | 42% | 8.2 |
| INCC-0256 | India^d^ | 49% | 8.2 |
| INMC-0504 | India^d^ | 50% | 8.8 |
| ETGA-0905 | Ethiopia^c^ | 55% | 6.1 |
| INMC-0505 | India^d^ | 56% | 13.4 |
| BRH1420534 | US^f^ | 57% | 7.0 |
| ETGA-1220 | Ethiopia^c^ | 57% | 8.4 |
| B82 | Bangladesh^a^ | 58% | 6.9 |
| ETGA-1034 | Ethiopia^c^ | 58% | 8.4 |
| ETGA-1285 | Ethiopia^c^ | 60% | 6.6 |
| ETGA-0981 | Ethiopia^c^ | 60% | 6.4 |
| INMC-0490 | India^d^ | 61% | 6.3 |
| INMC-0509 | India^d^ | 63% | 11.1 |
| INCC-0409 | India^d^ | 63% | 7.6 |
| USPA159 | US^e^ | 64% | 6.4 |
| B141 | Bangladesh^a^ | 67% | 8.7 |
| BRH1420533 | US^f^ | 67% | 8.7 |
| ETGA-1221 | Ethiopia^c^ | 69% | 7.8 |
| ETGA-0985 | Ethiopia^c^ | 69% | 7.2 |
| ETGA-1289 | Ethiopia^c^ | 69% | 6.2 |
| BRMA-0415 | Brazil^b^ | 70% | 6.4 |

* Data from Thailand study (Pal et al., 2019) has not been included.

a. Data published within [21].

b .Data published within [20].

c. Data published at Domingo [23].

d. Data published at Domingo [24].

e. Data published within [22].

f. Data published within [19].

**Supplementary Table 11. Contingency tables showing agreement in classification of anemia status between the STANDARD G6PD Test and the reference Complete Blood Count (CBC) T-Hb measurement for a) Capillary specimens and b) Venous specimens (excluding contrived)**

1. Capillary

|  | | **CBC** | | | |
| --- | --- | --- | --- | --- | --- |
|  |  | **Severe anemia** | **Moderate anemia** | **Non/mild anemia** | **Total** |
| **STANDARD**  **G6PD Test** | **Severe anemia** | 33 | 32 | 2 | 66 |
|  | **Moderate anemia** | 3 | 127 | 149 | 279 |
|  | **Non/mild anemia** | 0 | 33 | 1,888 | 1,921 |
|  | **Total** | 36 | 192 | 2,039 | 2,267 |

Percent agreement between CBC and the STANDARD G6PD Test was 90.3% [95% CI: 89.0–91.5].

1. Venous (excluding contrived)

|  | | **CBC** | | | |
| --- | --- | --- | --- | --- | --- |
|  |  | **Severe anemia** | **Moderate anemia** | **Non/mild anemia** | **Total** |
| **STANDARD**  **G6PD Test** | **Severe anemia** | 33 | 11 | 2 | 46 |
|  | **Moderate anemia** | 4 | 135 | 69 | 208 |
|  | **Non/mild anemia** | 0 | 49 | 2,051 | 2,100 |
|  | **Total** | 37 | 195 | 2,122 | 2,354 |

Percent agreement between CBC and the STANDARD G6PD Test was 94.3% [95% CI: 93.2–95.2].

**Supplementary Table 12. Contingency tables showing agreement in classification of anemia status between the HemoCue and the reference Complete Blood Count (CBC) T-Hb measurement for a) Capillary specimens and b) Venous specimens (excluding contrived).**

Note: participants have only been included in this analysis if there were available STANDARD G6PD results for the relevant specimen types.

1. Capillary

|  | | **CBC** | | | |
| --- | --- | --- | --- | --- | --- |
|  |  | **Severe anemia** | **Moderate anemia** | **Non/mild anemia** | **Total** |
| **HemoCue** | **Severe anemia** | 28 | 4 | 1 | 33 |
|  | **Moderate anemia** | 6 | 130 | 47 | 183 |
|  | **Non/mild anemia** | 2 | 58 | 1,972 | 2,032 |
|  | **Total** | 36 | 192 | 2,020 | 2,254 |

Percent agreement between CBC and the HemoCue was 94.6% [95% CI: 93.7 – 95.6].

1. Venous (excluding contrived)

|  | | **CBC** | | | |
| --- | --- | --- | --- | --- | --- |
|  |  | **Severe anemia** | **Moderate anemia** | **Non/mild anemia** | **Total** |
| **HemoCue** | **Severe anemia** | 32 | 3 | 3 | 38 |
|  | **Moderate anemia** | 4 | 133 | 16 | 153 |
|  | **Non/mild anemia** | 1 | 52 | 1,960 | 2,013 |
|  | **Total** | 37 | 188 | 1,979 | 2,024 |

Percent agreement between CBC and the HemoCue was 96.4% [95% CI: 95.6 – 97.2].

**Supplementary Table 13. Difference in mean hemoglobin concentration for the STANDARD G6PD Test and the HemoCue 201+ system, for a) pooled estimates, and b) study-specific estimates**

**A) Pooled**

| **Comparison** | **Index test sample type** | **Mean difference** | **Standard deviation** |
| --- | --- | --- | --- |
| HemoCue vs CBC | Capillary | 0.30 | 1.00 |
| HemoCue vs CBC | Venous | 0.35 | 0.80 |
| STANDARD G6PD vs CBC | Capillary | -0.06 | 1.33 |
| STANDARD G6PD vs CBC | Venous | 0.47 | 1.18 |

**B) Study-specific**

| **Study** | **Index test sample type** | **SD Biosensor *vs* CBC** | | **HemoCue *vs* CBC** | |
| --- | --- | --- | --- | --- | --- |
|  |  | **Mean difference** | **Standard deviation** | **Mean difference** | **Standard deviation** |
| Bangladesh | Venous | 0.47 | 0.74 | NA | NA |
| Brazil (Manaus) | Capillary | 0.50 | 1.17 | 0.75 | 0.83 |
| Brazil (Manaus) | Venous | 0.40 | 1.17 | 0.64 | 0.87 |
| Ethiopia | Capillary | 0.55 | 1.18 | 0.003 | 0.99 |
| Ethiopia | Venous | 0.82 | 1.32 | 0.02 | 0.73 |
| India | Capillary | -0.86 | 1.10 | -0.10 | 1.03 |
| India | Venous | 0.43 | 1.20 | 0.18 | 0.70 |
| Thailand | Venous | -0.57 | 0.77 | NA | NA |
| US: Contrived | Venous | 0.45 | 0.48 | NA | NA |
| US: Pennsylvania | Capillary | -0.99 | 0.91 | 0.37 | 0.90 |
| US: Pennsylvania | Venous | 0.78 | 1.28 | 0.45 | 0.85 |
| US: Washington | Capillary | -1.05 | 1.01 | -0.12 | 0.93 |
| US: Washington | Venous | 0.43 | 0.90 | 0.15 | 0.24 |

**Supplementary Table 14. 3x3 agreement tables between the STANDARD G6PD Test and the reference assay G6PD percent activity, on capillary specimens for A. males, and B. females.**

**A. Males**

|  | | **Reference assay % activity** | | | **TOTAL** |
| --- | --- | --- | --- | --- | --- |
|  |  | **≤30%** | **30-70%** | **>70%** |  |
| **STANDARD G6PD Test** | ≥6.1 U/g Hb | 0 | 23 | 1830 | 1853 |
|  | 6-4 U/g Hb | 0 | 15 | 153 | 168 |
|  | ≤ 4 U/g Hb | 134 | 11 | 16 | 161 |
|  | **TOTAL** | 134 | 49 | 1999 | 2182 |

**B. Females**

|  | | **Reference assay % activity** | | | **TOTAL** |
| --- | --- | --- | --- | --- | --- |
|  |  | **≤30%** | **30-70%** | **>70%** |  |
| **STANDARD G6PD Test** | ≥6.1 U/g Hb | 9 | 30 | 19 | 58 |
|  | 6-4 U/g Hb | 0 | 37 | 120 | 157 |
|  | ≤ 4 U/g Hb | 0 | 20 | 1795 | 1815 |
|  | **TOTAL** | 9 | 87 | 1934 | 2030 |
