# Supplementary material for: Clinical performance validation of the STANDARD G6PD test: A multi-country pooled analysis
Source: PLoS Negl Trop Dis. 2023 Oct 12;17(10):e0011652. doi: 10.1371/journal.pntd.0011652 (PMC10597494; doi:10.1371/journal.pntd.0011652)
Supplement: S11 Table — (DOCX) [file pntd.0011652.s011.docx]

**S11 Table. Contingency tables showing agreement in classification of anemia status between the STANDARD G6PD Test and the reference Complete Blood Count (CBC) T-Hb measurement for a) Capillary specimens and b) Venous specimens (excluding contrived)**

1. Capillary

|  | | **CBC** | | | |
| --- | --- | --- | --- | --- | --- |
|  |  | **Severe anemia** | **Moderate anemia** | **Non/mild anemia** | **Total** |
| **STANDARD**  **G6PD Test** | **Severe anemia** | 33 | 32 | 2 | 66 |
|  | **Moderate anemia** | 3 | 127 | 149 | 279 |
|  | **Non/mild anemia** | 0 | 33 | 1,888 | 1,921 |
|  | **Total** | 36 | 192 | 2,039 | 2,267 |

Percent agreement between CBC and the STANDARD G6PD Test was 90.3% [95% CI: 89.0–91.5].

1. Venous (excluding contrived)

|  | | **CBC** | | | |
| --- | --- | --- | --- | --- | --- |
|  |  | **Severe anemia** | **Moderate anemia** | **Non/mild anemia** | **Total** |
| **STANDARD**  **G6PD Test** | **Severe anemia** | 33 | 11 | 2 | 46 |
|  | **Moderate anemia** | 4 | 135 | 69 | 208 |
|  | **Non/mild anemia** | 0 | 49 | 2,051 | 2,100 |
|  | **Total** | 37 | 195 | 2,122 | 2,354 |

Percent agreement between CBC and the STANDARD G6PD Test was 94.3% [95% CI: 93.2–95.2].
