# Supplementary material for: Clinical performance validation of the STANDARD G6PD test: A multi-country pooled analysis
Source: PLoS Negl Trop Dis. 2023 Oct 12;17(10):e0011652. doi: 10.1371/journal.pntd.0011652 (PMC10597494; doi:10.1371/journal.pntd.0011652)
Supplement: S12 Table — (DOCX) [file pntd.0011652.s012.docx]

**S12 Table. Contingency tables showing agreement in classification of anemia status between the HemoCue and the reference Complete Blood Count (CBC) T-Hb measurement for a) Capillary specimens and b) Venous specimens (excluding contrived).**

Note: participants have only been included in this analysis if there were available STANDARD G6PD results for the relevant specimen types.

1. Capillary

|  | | **CBC** | | | |
| --- | --- | --- | --- | --- | --- |
|  |  | **Severe anemia** | **Moderate anemia** | **Non/mild anemia** | **Total** |
| **HemoCue** | **Severe anemia** | 28 | 4 | 1 | 33 |
|  | **Moderate anemia** | 6 | 130 | 47 | 183 |
|  | **Non/mild anemia** | 2 | 58 | 1,972 | 2,032 |
|  | **Total** | 36 | 192 | 2,020 | 2,254 |

Percent agreement between CBC and the HemoCue was 94.6% [95% CI: 93.7 – 95.6].

1. Venous (excluding contrived)

|  | | **CBC** | | | |
| --- | --- | --- | --- | --- | --- |
|  |  | **Severe anemia** | **Moderate anemia** | **Non/mild anemia** | **Total** |
| **HemoCue** | **Severe anemia** | 32 | 3 | 3 | 38 |
|  | **Moderate anemia** | 4 | 133 | 16 | 153 |
|  | **Non/mild anemia** | 1 | 52 | 1,960 | 2,013 |
|  | **Total** | 37 | 188 | 1,979 | 2,024 |

Percent agreement between CBC and the HemoCue was 96.4% [95% CI: 95.6 – 97.2].
