# Supplementary material for: Clinical performance validation of the STANDARD G6PD test: A multi-country pooled analysis
Source: PLoS Negl Trop Dis. 2023 Oct 12;17(10):e0011652. doi: 10.1371/journal.pntd.0011652 (PMC10597494; doi:10.1371/journal.pntd.0011652)
Supplement: S13 Table — (DOCX) [file pntd.0011652.s013.docx]

**S13 Table. Difference in mean hemoglobin concentration for the STANDARD G6PD Test and the HemoCue 201+ system, for a) pooled estimates, and b) study-specific estimates**

**A) Pooled**

| **Comparison** | **Index test sample type** | **Mean difference** | **Standard deviation** |
| --- | --- | --- | --- |
| HemoCue vs CBC | Capillary | 0.30 | 1.00 |
| HemoCue vs CBC | Venous | 0.35 | 0.80 |
| STANDARD G6PD vs CBC | Capillary | -0.06 | 1.33 |
| STANDARD G6PD vs CBC | Venous | 0.47 | 1.18 |

**B) Study-specific**

| **Study** | **Index test sample type** | **SD Biosensor *vs* CBC** | | **HemoCue *vs* CBC** | |
| --- | --- | --- | --- | --- | --- |
|  |  | **Mean difference** | **Standard deviation** | **Mean difference** | **Standard deviation** |
| Bangladesh | Venous | 0.47 | 0.74 | NA | NA |
| Brazil (Manaus) | Capillary | 0.50 | 1.17 | 0.75 | 0.83 |
| Brazil (Manaus) | Venous | 0.40 | 1.17 | 0.64 | 0.87 |
| Ethiopia | Capillary | 0.55 | 1.18 | 0.003 | 0.99 |
| Ethiopia | Venous | 0.82 | 1.32 | 0.02 | 0.73 |
| India | Capillary | -0.86 | 1.10 | -0.10 | 1.03 |
| India | Venous | 0.43 | 1.20 | 0.18 | 0.70 |
| Thailand | Venous | -0.57 | 0.77 | NA | NA |
| US: Contrived | Venous | 0.45 | 0.48 | NA | NA |
| US: Pennsylvania | Capillary | -0.99 | 0.91 | 0.37 | 0.90 |
| US: Pennsylvania | Venous | 0.78 | 1.28 | 0.45 | 0.85 |
| US: Washington | Capillary | -1.05 | 1.01 | -0.12 | 0.93 |
| US: Washington | Venous | 0.43 | 0.90 | 0.15 | 0.24 |
