# Supplementary material for: Clinical performance validation of the STANDARD G6PD test: A multi-country pooled analysis
Source: PLoS Negl Trop Dis. 2023 Oct 12;17(10):e0011652. doi: 10.1371/journal.pntd.0011652 (PMC10597494; doi:10.1371/journal.pntd.0011652)
Supplement: S14 Table — (DOCX) [file pntd.0011652.s014.docx]

**S14 Table. 3x3 agreement tables between the STANDARD G6PD Test and the reference assay G6PD percent activity, on capillary specimens for A. males, and B. females.**

**A. Males**

|  | | **Reference assay % activity** | | | **TOTAL** |
| --- | --- | --- | --- | --- | --- |
|  |  | **≤30%** | **30-70%** | **>70%** |  |
| **STANDARD G6PD Test** | ≥6.1 U/g Hb | 0 | 23 | 1830 | 1853 |
|  | 6-4 U/g Hb | 0 | 15 | 153 | 168 |
|  | ≤ 4 U/g Hb | 134 | 11 | 16 | 161 |
|  | **TOTAL** | 134 | 49 | 1999 | 2182 |

**B. Females**

|  | | **Reference assay % activity** | | | **TOTAL** |
| --- | --- | --- | --- | --- | --- |
|  |  | **≤30%** | **30-70%** | **>70%** |  |
| **STANDARD G6PD Test** | ≥6.1 U/g Hb | 9 | 30 | 19 | 58 |
|  | 6-4 U/g Hb | 0 | 37 | 120 | 157 |
|  | ≤ 4 U/g Hb | 0 | 20 | 1795 | 1815 |
|  | **TOTAL** | 9 | 87 | 1934 | 2030 |
