# Supplementary figures and images for: Clinical performance validation of the STANDARD G6PD test: A multi-country pooled analysis
Source: PLoS Negl Trop Dis. 2023 Oct 12;17(10):e0011652. doi: 10.1371/journal.pntd.0011652 (PMC10597494; doi:10.1371/journal.pntd.0011652)

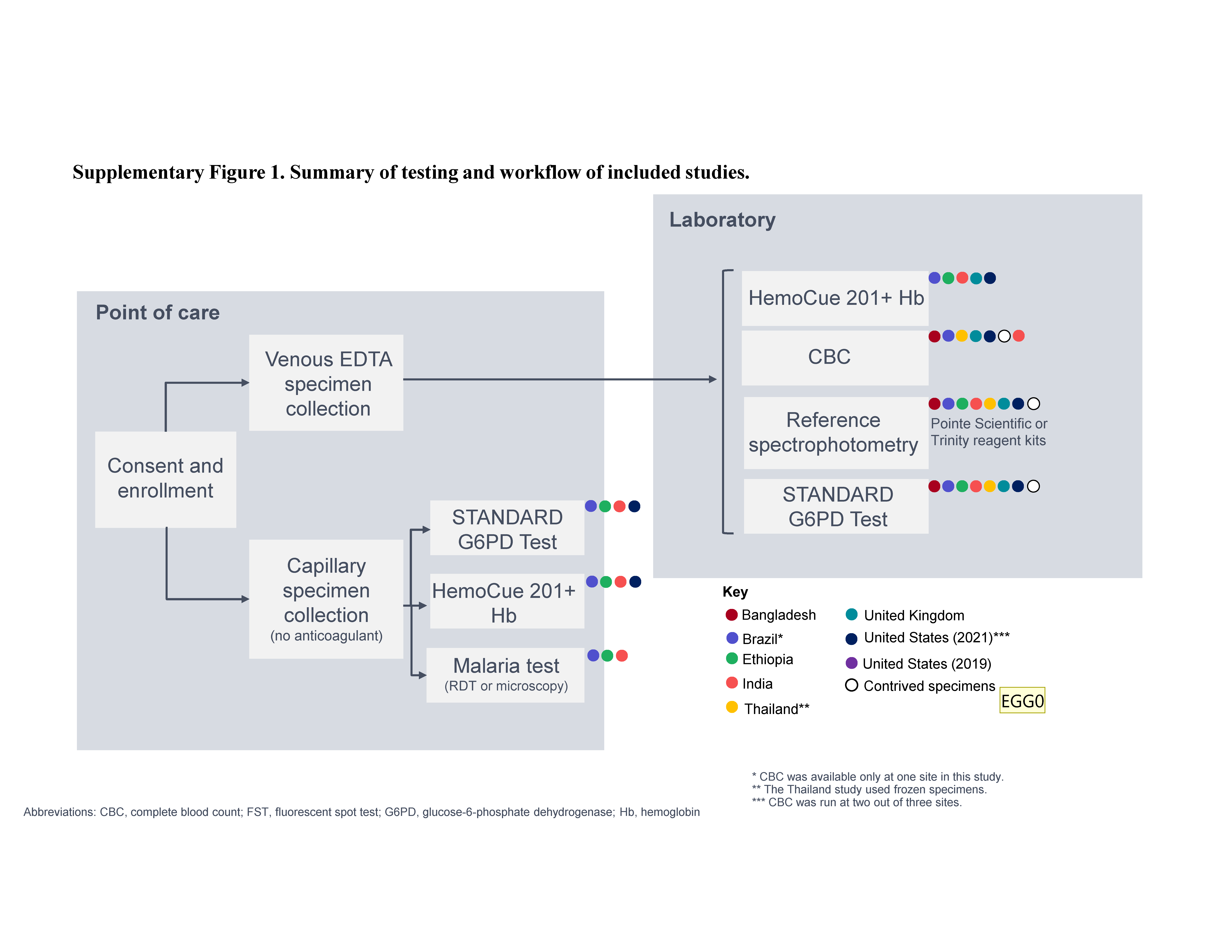

Supplement: S1 Fig — (TIFF) [file pntd.0011652.s015.tiff]

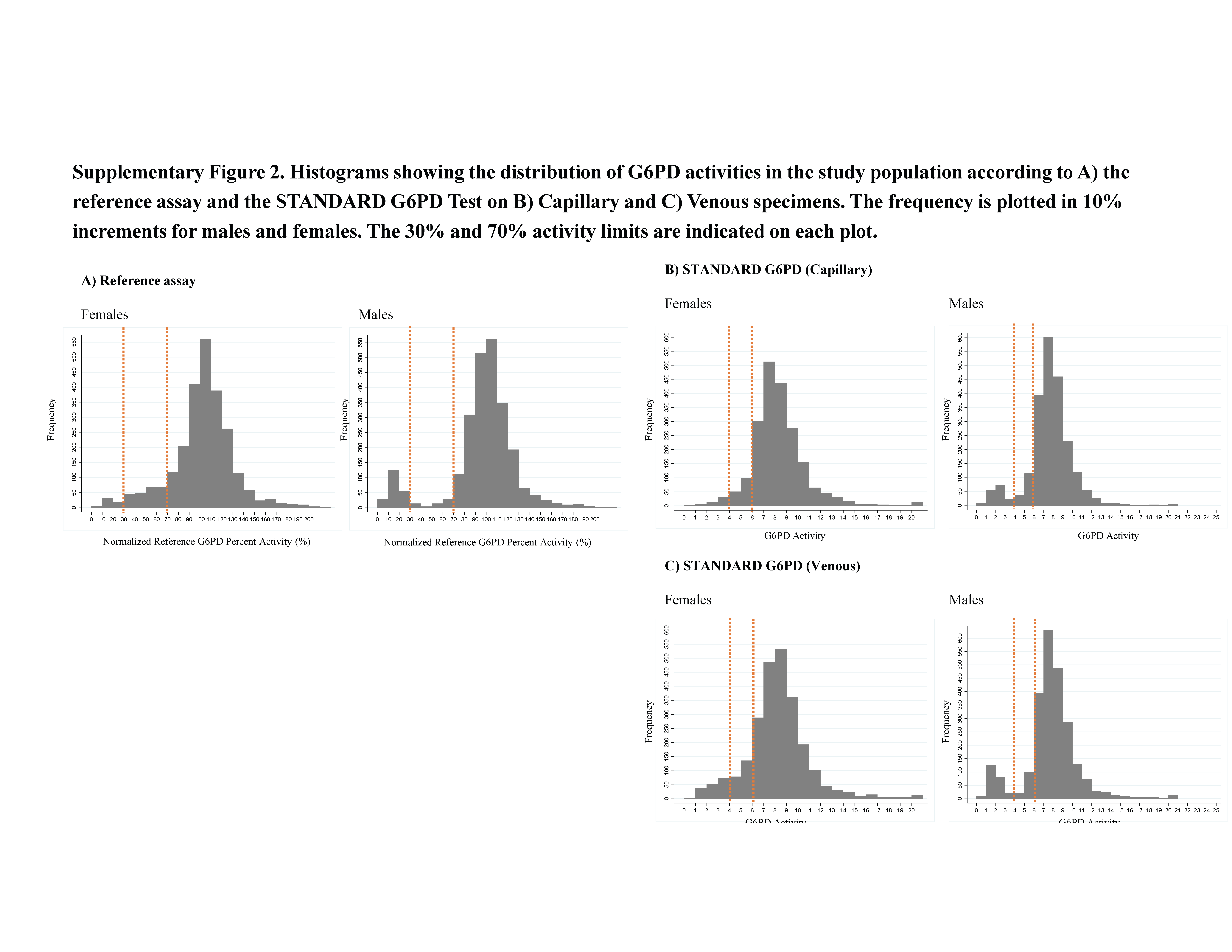

Supplement: S2 Fig — The frequency is plotted in 10% increments for males and females. The 30% and 70% activity limits are indicated on each plot. (TIFF) [file pntd.0011652.s016.tiff]

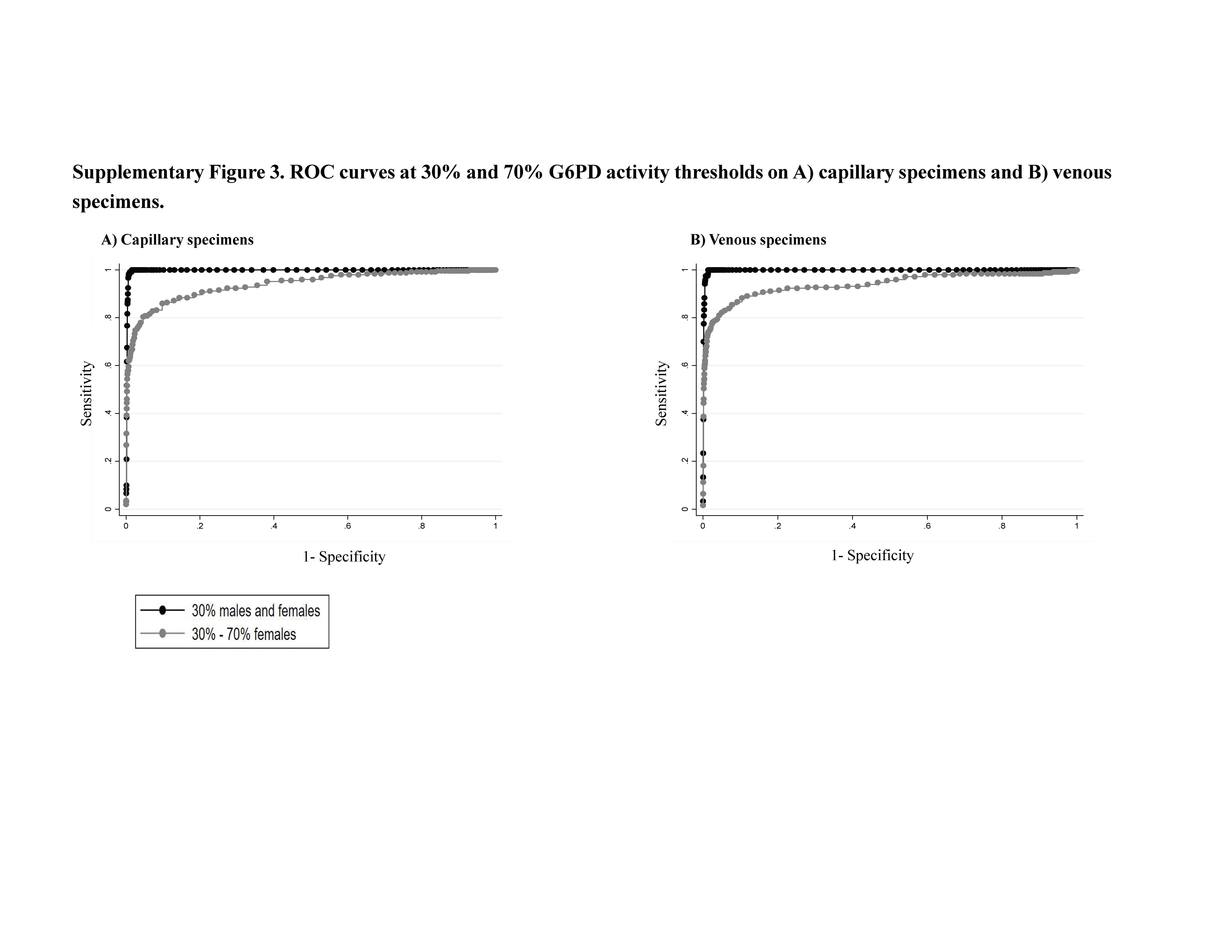

Supplement: S3 Fig — (TIFF) [file pntd.0011652.s017.tiff]

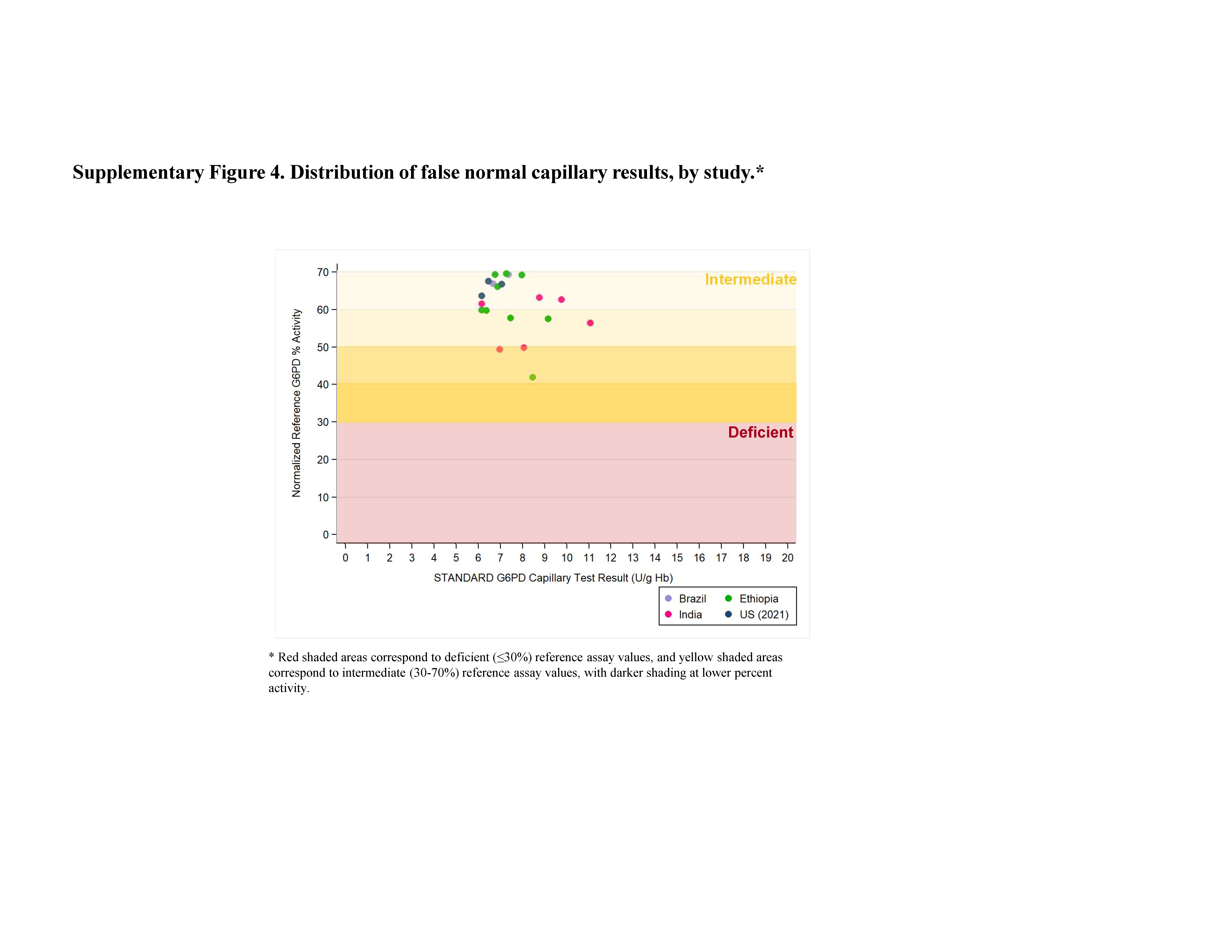

Supplement: S4 Fig — (TIFF) [file pntd.0011652.s018.tiff]

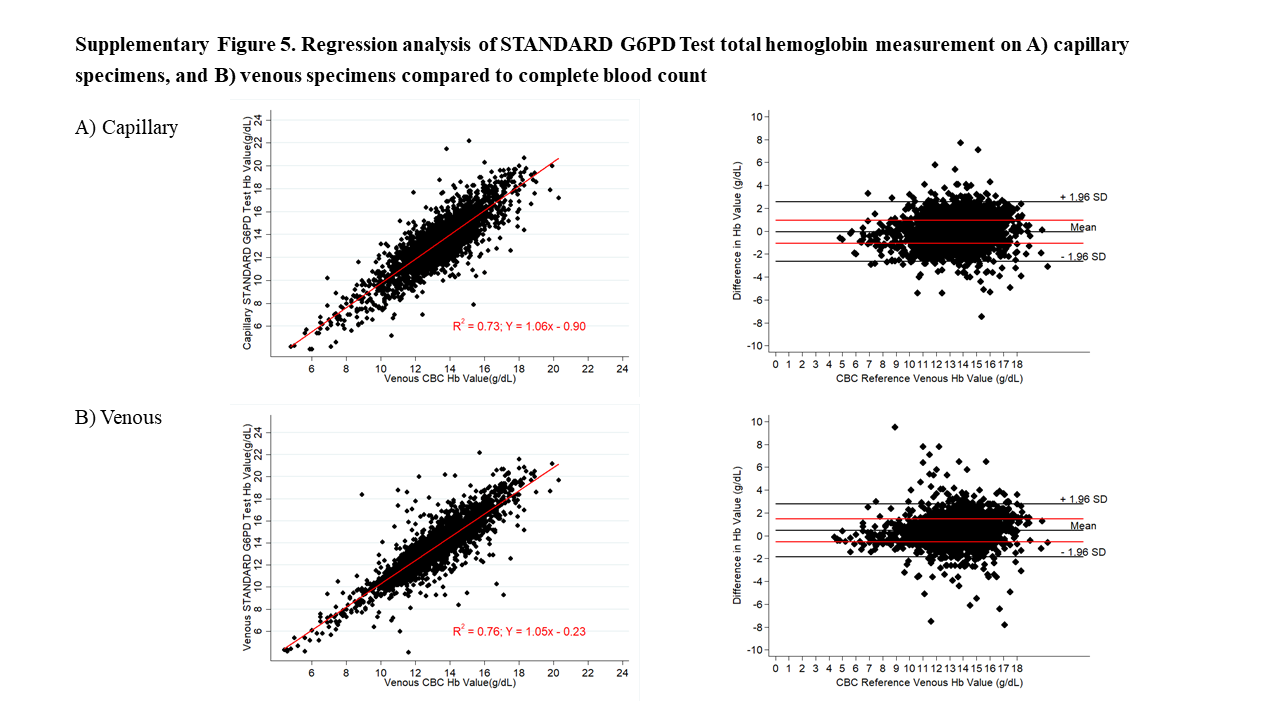

Supplement: S5 Fig — (TIF) [file pntd.0011652.s019.tif]

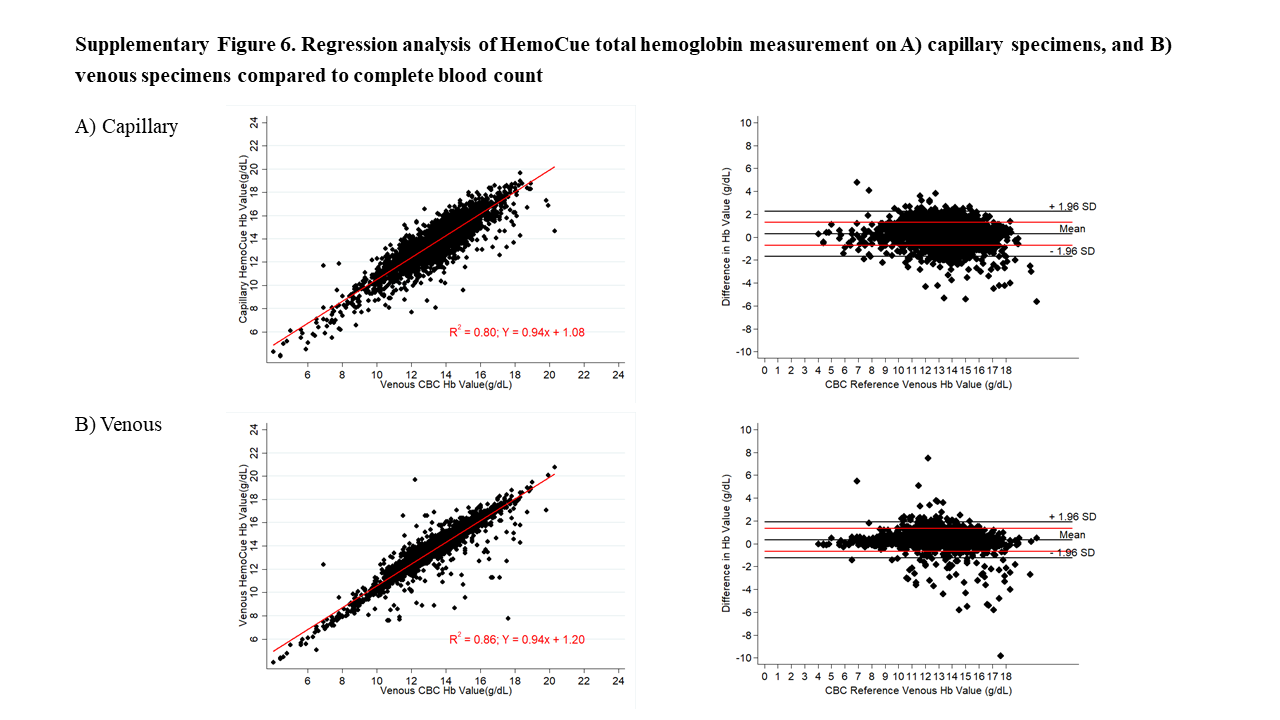

Supplement: S6 Fig — (TIF) [file pntd.0011652.s020.tif]
